# Occurrence and removal characteristics of phthalate esters from bottled drinking water using silver modified roasted date pits

**DOI:** 10.1007/s40201-021-00642-9

**Published:** 2021-03-20

**Authors:** Fedae A. Alhaddad, Mohammed Abu-Dieyeh, Dana Da’ana, Murad Helaleh, Mohammad A. Al-Ghouti

**Affiliations:** 1 Department of Biological and Environmental Sciences, College of Arts and Sciences, Qatar University, P.O. Box 2713, State of Qatar Doha,; 2 Department of Biology and Biotechnology, Faculty of Science, The Hashemite University, P.O. Box 330127, Zarqa, 13133 Jordan; 3 Section Head Supplements Testing, Anti Doping Lab Qatar, P.O. Box 27775, Doha, Qatar

**Keywords:** Phthalate esters, Adsorption, Bottled drinking water, Date pits, Silver modified roasted date pits

## Abstract

**Background:**

This paper aims to investigate the occurrence and removal characteristics of phthalate esters from bottled drinking water using silver modified roasted date pits. Three adsorbents, namely roasted date pits (RODP), silver-modified roasted date pits (S-RODP), and activated carbon (AC) were used to investigate their adsorption characterizations in removing dimethyl phthalate (DMP), diethyl phthalate (DEP), dibutyl phthalate (DBP), butyl benzyl phthalate (BBP), di-2-ethylhexyl phthalate (DEHP), and di-n-octyl phthalate (DNOP) from the collected bottle water samples.

**Methods:**

The occurrences of the phthalate esters in the collected bottled water samples were carried out at different temperatures (30, 50, and 60 °C), and analyzed using gas chromatography-mass spectrometry analysis - selected ion monitoring. Batch adsorption isotherms were used to study and establish the efficiency of such adsorbents in removing phthalate esters, in which they describe the adsorbent-adsorbate interaction systems. Adsorption efficiency of the various adsorbents was investigated by using different adsorbent masses (0.05 g, 0.10 g, and 0.15 g) and temperature (30 °C, 50 °C, and 60 °C). Different physical and chemical characterizations were studied using scanning electron microscopy (SEM), Fourier transform infrared (FTIR), Brunauer-Emmett-Teller (BET) surface area, pore radius, and pore volume.

**Results:**

The results indicated that the most abundant phthalate esters were DMP followed by DEP under 30 °C; however, DNOP was not detected in any of the tested water samples, except for one sample under 30 °C with a concentration of 0.031 μg/mL. The obtained results showed that phthalate esters leaching to the bottled drinking water were affected by storage temperature. The phthalate esters levels were increased with increasing the temperature to 60 °C. It was concluded that the ability of S-RODP for the adsorption of phthalate esters was better than the removal percentage obtained by AC and RODP. The removal percentage was increased from 90 to 99% by increasing the temperature from 30 to 50 °C and then decreased to 92.3% at 60 °C.

**Conclusion:**

RODP was successfully used as an effective adsorbent for phthalate esters removal from drinking water. However, S-RODP has the highest removal abilities than other adsorbents due to the newly formed functional groups on its surface.

## Introduction

The increased consumption of plastics in our daily lives is growing up. According to Laville and Taylor [25], around a million plastic bottles are being used worldwide each minute and by 2021, the usage of plastic bottles will increase by 20%. Polyethylene terephthalate (PET) is a recyclable polymer that is the most commonly used for bottling of drinking water, however, in 2016, only 7% of the collected plastic bottles were recycled into new bottles and the rest ended up in the ocean or landfill. According to Ellen MacArthur Foundation [13], the annual leaching of plastic into the world’s ocean is in the range of 5–13 million tons, which are ingested by aquatic organisms. Experts say that if it continued to be in that state, by 2050 there will be more plastic by weight in the ocean than fishes.

Nowadays, PET is the most widespread material for the production of water bottles, as the world witnessed a surge in the usage of PET bottled water due to the low production cost, ease of transport, as well as its size, and strength. As a result, the migration of phthalate from PET water bottles formed a global concern [22]. The variation in the phthalate forms could be attributed to the addition of alkyl groups [27]. Phthalates are widely used in various industrial applications, plastic products, production of food cans, production of personal-caring products, food wrap, packing automotive parts, and many other products [40]. Even though exposure to excessive amounts of phthalates can lead to harmful and diverse effects (e.g. toxicities, carcinogenic threat, allergies, and others), they are still being used widely in personal products. Hence, many national and international organizations have set some rules on the usage of phthalates that must go under close supervision [36]. Furthermore, the source of phthalate in plastic bottled water had gained an increased concern. It either could be present from the recycling of PET plastics, water resources before bottling or leached from the bottle material into the water [18]. Moreover, Elobeid et al. [14] suggested that the migration of phthalates that occurs during the storage process could be due to the degradation of organic compounds or photolytic formation. Al-Saleh et al. [5] investigated the presence of phthalates in different bottled water brands stored under different conditions. It was shown that phthalates were present in all tested water samples regardless of the storing conditions. Regarding the phthalates solubility in water, dimethyl phthalate (DMP) has the highest solubility among other commonly used phthalate esters of 1080 μg/mL, followed by diethyl phthalate (DEP) of 1000 μg/mL. The solubility for butyl benzyl phthalate (BBP) and bis(2-ethylhexyl) phthalate (DEHP) are 2.69 and 0.3 μg/mL, respectively, while di-n-octyl phthalate (DNOP) is water-insoluble [26]. Various organizations had set certain limits for the concentrations and levels of phthalates in drinking water due to their harmful effect on human health. According to the EU Council [17], the DEHP and BBP maximum contaminant levels (MCL) are 0.006 μg/mL and 1 × 10^−4^ μg/mL, respectively. While the threshold limit values (TLV) for DEP, DBP, DMP, and DEHP are 0.55 μg/mL, 0.45 μg/mL, 5.0 μg/mL, and 5.0 μg/mL, respectively.

In the literature, there are various methods for the removal of phthalates from contaminated water [28, 42]. Phthalate treatment from water can be done by physiochemical, biological, and advanced oxidation processes. The biological treatment process can be done through degradation, hydrolysis, and photolysis. However, these methods are slow and take longer time than do biodegradation. According to Xu et al. [42], several studies showed that the degradation of phthalates in the aquatic system by microbial activity is the major degradation mechanism. Moreover, the physicochemical treatment process includes floatation and coagulation/flocculation to reduce the suspended solids, colloidal particles, and floating materials.

However, the common disadvantage for most existing treatment technologies is lower efficiency and longer treatment period, in addition to the operational and maintenance costs [28]. According to the USA Environmental Protection Agency (EPA), the use of activated carbon (AC) as an adsorbent is considered one of the oldest practices and the most efficient methods for the removal of organic pollutants from water [28]. Here, the ease of operation, simplicity of design, and high removal efficiency (90–99%) are advantages of adsorption over other techniques. Adsorption may involve π-complexation, van der Waals, and electrostatic interactions or chemisorption. Furthermore, AC is the most common adsorbent used for pollutants removal and wastewater treatment, but its high cost limits its usage and increases the need for an alternative adsorbent. According to Shaida et al. [37], various adsorbent types of nanostructured materials can be used for the sorption of phthalates with high adsorption capacities, but their use is challenging due to the disposal of these materials after adsorption and having limitations in synthesizing them in large quantities. As a result, using low cost and naturally derived adsorbents are more relevant. Agricultural wastes such as date pits (DPs) are considered as cost-effective adsorbents compared to AC, as DPs have macrostructure, physical and chemical properties such as insolubility in water, high mechanical strength, chemical stability, economic viability, and zero economic value and approximately forms 15% of date fruit weight [3]. The chemical composition of DPs on a dry matter basis was cellulose: 21.2 ± 0.1, hemicelluloses: 28.1 ± 0.1; and lignin: 19.9 ± 0.1%wt [4]. The adsorption properties of DPs can be enhanced by the impregnation of some metal ions such as silver ions. In this respect, the metal oxide may possess many advantages in adsorption of phthalates from water; allowing phthalates molecules to readily penetrate their structures and be removed easily, namely by acid-base properties, pore structure, high surface area, and pore volume.

This paper aims to investigate the presence of phthalate esters in bottled water from different bottled water brands using gas chromatography-mass spectroscopy (GC-MS). Furthermore, silver nitrate was used to modify the roasted date pits (RODP) to produced silver-modified roasted date pits (S-RODP). In this study, the three adsorbents (RODP, S-RODP, and AC) were used to investigate their adsorption characterizations in removing dimethyl phthalate (DMP), diethyl phthalate (DEP), dibutyl phthalate (DBP), di-2-Ethylhexyl phthalate (DEHP), and di-n-octyl phthalate (DNOP) from different bottle water samples. Batch adsorption isotherms were used to study and establish the effectiveness and capacity of such adsorbents in removing phthalate esters is in which they describe the adsorbent-adsorbate relationship in an aqueous medium at a constant temperature [4].

## Materials and methods

### Samples collection and storage conditions

Six various brands of bottled water samples (A, D, E, H, Q, and R) were randomly collected from local mini, super, and hypermarkets in Doha, Qatar, which are locally and internationally produced and frequently consumed. All sampled bottled waters were of the same size (1.5 L), and all samples were packed within PET plastic bottles. All samples were stored inside the markets with the common storage conditions of bottled waters in retail outlets and supermarkets; i.e. the samples were not exposed to sunlight once the sampling occurred. All sampled bottled waters were of the same size (1.5 L), and all samples were packed within PET plastic bottles. The locally produced water bottled samples were named D and Q while the international bottled samples were named as A, E, and H. Bottled water samples were analyzed after being stored in the oven for 48 h at different temperatures (30, 50, and 60 °C). Each sample was analyzed in duplicates and the duplicate analysis values were averaged.

### Preparation of stock standards

#### Internal standards stock solution

The internal standards used in the analysis were obtained from Cambridge Isotope Laboratories, Inc. as follows: EPA 267B, butyl benzyl phthalate (99%), 5000 μg/mL; EPA 266B, bis(2-ethylhexyl) phthalate (99%), 5000 μg/mL; and EPA 271B, dimethyl phthalate (99%), 5000 μg/mL.

#### Preparation of phthalate stock standard

A 1000 μg/mL was transferred into a 10 mL volumetric flask, and then it was diluted with hexane to produce a 100 μg/mL stock solution. The 10 mL stock solution was divided into 10 aliquots, each 1 mL was capped using a crimper cap and stored in the refrigerator until it is used. The remaining 0.2 mL was transferred into a 1 mL vial capped using a crimper cap and stored in the refrigerator until it is used.

#### Preparation of internal standards stock solution

Around 1/3 volume of hexane into 1 mL was added to class A volumetric flask, then 200 μL of phthalate internal standard solution was added using an appropriate syringe with mixing and made up to volume (5 mL) with hexane.

#### Preparation of phthalate calibration standards

The stock standard was used to prepare the calibration standards. Using an appropriate syringe, the calibration working solutions were prepared. The working calibration curves were verified based on the following: the phthalates final concentration were ranged from 0.00 to 60.0 ng/mL, the semi-volatile organic compounds (SVOCs) volumes were 5, 10, 20, 40, 60, 25, 5, 100, and 250 μL, the SVOC concentrations were 1.0 μg/mL and 100 μg/mL, the internal standard (ISTD) concentration was 5 μg/mL, and the ISTD volume of 40 μg/mL was 12.5 μL.

### Phthalate extraction procedure

The methodology employed in this paper is adopted from a well-established methodology used previously [32], and it is as follows: phthalate calibration standards with internal standards and surrogates were prepared. 100 mL of each water sample was transferred into a 250 mL separatory funnel after adjusting its pH to more than 11 by 6 M NaOH and then 10 mL dichloromethane (DCM) was added to each separatory funnel. After that, the quality control samples (QC1 and QC2), blank, and water samples were spiked with 12.5 μL SVOCs internal standard (40 μg/mL) in a separatory funnel. The separatory funnel was then shaken by an auto shaker 3 times each for 2 min with frequent venting to release the pressure, each at 80 rpm, allowing the DCM layer to settle for 10 min and then decant the layer into a collection bottle. The extraction steps were repeated two more times to ensure that all the analytes are recovered, the extract was collected as a base/neutral portion. After that, sodium sulfate was added until it forms a cake to remove the water from the DCM extract; the total extract when pooled should be more than 30 mL. The extract was transferred into a test tube and a rotary evaporator (40–50 °C) was used to concentrate the extract to 1–2 mL by a stream of nitrogen (dryness should be avoided). After that, the extract was further dried using Turbo-Vab (40 °C, 5 psi) until reaching a volume of 200 μL. Finally, the internal standards, samples, and blanks were loaded into the GC auto-sampler.

### Quantitation and quality control

The concentrations of phthalates were calculated using ratios calibration curves based on the peak area of native phthalates over the deuterated phthalates peak area. The deuterated was plotted relying on the ratios of native concentration of phthalates over the concentration of deuterated, as follows: for the DMP, DEP, DBP, DNOP, DEHP, the standard used was the deuterated standard; while for DiBP, the DEP-d_4_ was used. The DEHP-d_4_ was used for the DHP, DEHP, and BBP.

The identities of the phthalates were confirmed based on the retention time compared to that of standard (e.g. within ±0.01 min) and being within ±30% of the ratios of at least one of the two ions compared to the standard. It was noticed from the literature that few phthalates were also reported in the de-ionized water at a level comparable to ones found in bottled water samples. To overcome this issue, the blank deionized water was measured before performing the experiments and the average levels of phthalates in blanks were used to adjust the results of phthalate found in standard solutions in water by subtracting it. The same principle has been applied to the fiber blanks exposed to the same conditions as both the standards and samples.

#### Quality control standard **(**QCS) / percent recovery calculations

The recovery of each spike compound into the deionized water sample and the concentration of each spike compounds were calculated using the eq. 1:

1 $$ Spike\ Recovery=\left[\frac{SSR- SR}{SA}\right]x10 $$

Where SSR is the calculated concentration of the spiked sample, SR is the sample result (non-spiked), and SA is the spike concentration added.

### Instrument conditions and settings

Agilent gas chromatograph 7890A and 7693 Autosampler equipped with a 5975C mass spectrometer with triple-axis Detector was applied for GC-MS analysis using selected ion monitoring (SIM). The mass spectrometer was operated in electron impact mode (70 eV) at an emission current of 60 mA (solvent delay: 3.1 min; scan range: 40–500 m/z; ion source temperature: 280 °C; quadrupole temperature: 180 °C; acquisition mode: SIM-selected ion monitoring, and ionization: electron impact). One microliter of the sample extract into the injection port of the GC. The autosampler was set to rinse a minimum of three times with hexane before and after sample injection. Helium with a purity of 99.999% was used as the carrier gas at a flow rate of 10 mL/min, and separation of analyte was attained with HP-5 capillary column (30 m × 0.25 mm ID × 0.25 μm film thickness; 1.0 mL/min (constant pressure); 50 mL/min (split ratio = 50:1). The initial oven temperature was set at 40 °C with a holding time of 2 min then raised to 290 °C at a rate of 15 °C/min and increased to 303 °C at a rate of 2 °C/min and further increased to 320 °C at a rate of 4 °C/min held for 3 min. The ion source and interface temperatures were set at 230 °C and 300 °C, respectively. The range of mass ion (m/z) was 50–500. Scanning interval and selected ion monitoring (SIM) sampling rate were 0.5 and 0.2 s, respectively. The mass selective detector was operated in SIM mode by monitoring three mass ions for phthalates (Dimethyl phthalate_D4 (ISTD), RT: 11.609, Quant. Ion (m/z): 167, Qual. Ion (m/z): 81; DMP, RT: 11.615, Quant. Ion (m/z): 163, Qual. Ion (m/z): 77; DEP, RT: 12.843, Quant. Ion (m/z): 149, Qual. Ion (m/z): 177; DBP, RT: 15.676, Quant. Ion (m/z): 149, Qual. Ion (m/z): 104; Benzyl butyl phthalate_D4 (ISTD), RT: 18.234, Quant. Ion (m/z): 153, Qual. Ion (m/z): 91; BBP, RT: 18.250, Quant. Ion (m/z): 149, Qual. Ion (m/z): 91; Bis(2-ethylhexyl) phthalate_D4 (ISTD), RT: 19.319, Quant. Ion (m/z): 153, Qual. Ion (m/z): 171; DEHP, RT: 19.325, Quant. Ion (m/z): 149, Qual. Ion (m/z): 167; and DNOP, RT: 20.547, Quant. Ion (m/z): 149, Qual. Ion (m/z): 167).

### Silver-modified roasted date pits (S-RODP) preparation procedure

**Fig. 1:**
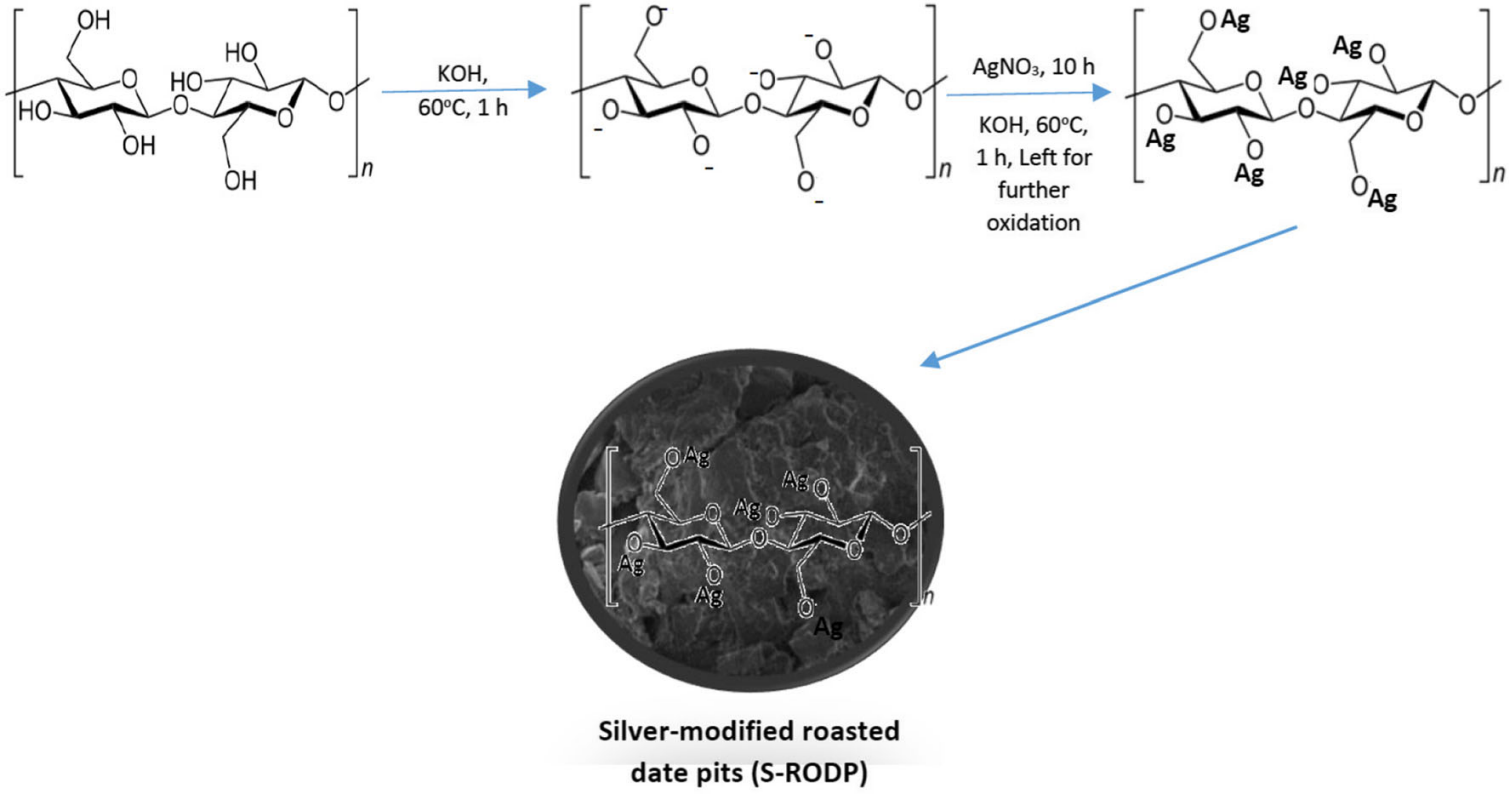
S-RODP preparation

The raw date pits (RDP) were obtained from local markets in Doha, Qatar. The dried RDP was then roasted at 130 °C for 3 h in an oven to produce roasted date pits (RODP). The RODP was grounded into particles size ranging from coarse particles to fine particles. One particle size range (0.250 mm - 0.125 mm) was used. Silver-RODP (S-RODP) was then prepared by reacting 10 g of RODP and 5.61 g KOH with 100 mL distilled water for 1 h at a temperature of 60 °C. Then, 1.698 g of AgNO_3_ was added to the solution for 10 h [33]. After that, the resulting solid was reacted with 5.61 g KOH for 10 h, and then they were left for further oxidation as shown in Fig. 1. The formed solid, which is S-RODP, was washed with plenty of water, dried at 105 °C, and stored in glass bottles. Activated carbon (AC) (Sigma Aldrich, CAS No. 7440-44-0) that is locally available was used as a reference material due to it is widely use in the remediation and removal applications of different pollutants. Table 1 shows the BET isothermal analysis for the adsorbents.

**Table 1:** BET isothermal analysis

Adsorbents	BET Surface Area (m^2^/g)	Langmuir Surface Area (m^2^/g)	Total Pore Volume (cm^3^/g)	Average Pore Radius (Å)	Reference
RODP	3.184	4.039	0.009	55.0	Current study
S-RODP	3.009	3.991	0.008	54.0	Current study
AC	467.62	–	0.33	–	[19]

### Adsorption process

The batch adsorption experiments were performed under a fixed volume (100 mL) of each water sample (pH = 8) under different masses (0.05 g, 0.1 g, and 0.15 g) of each adsorbent (AC, RODP, and S-RODP) and temperatures (30 °C, 50 °C, and 60 °C). The batch adsorption experiments were conducted in 250 mL lidded glass beakers, each with 100 mL of the water sample and 0.05 g of the adsorbent (AC, RODP, and S-RODP). To ensure quality control and no experimental errors, two trials, and blanking of each batch experiment were conducted. The batch experiments were conducted using an incubator shaker (Shaking Incubator, MODEL: SSI10R-2, Orbital-Shaking, a temperature-controlled shaker) under a constant speed of 165 rounds per minute (rpm) for 72 h (the time needed to reach equilibrium). All samples were then filtered, and the phthalates were extracted from the residual solution based on the procedure described in section 2.5. After that, the phthalate concentration was determined using the GC-MS.

### Statistical analysis

Means and standard deviation of the final concentrations of different phthalates including (DMP, DEP, DBP, DEHP, & DNOP) in the bottled drinking water were calculated. The *P* value of less than 0.05 was considered as an indication of the statistical significance.

## Results and discussion

**Fig. 2:**
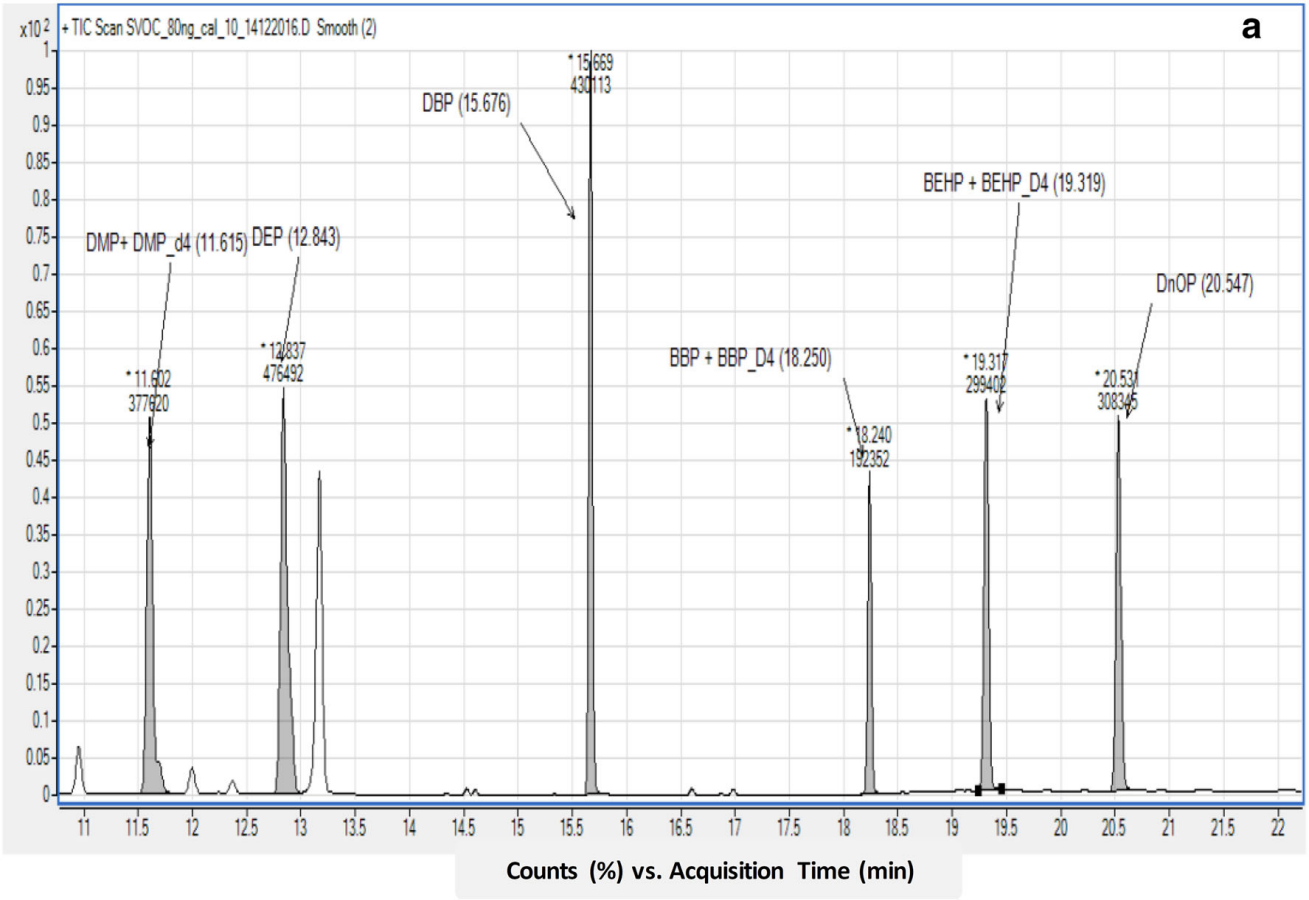

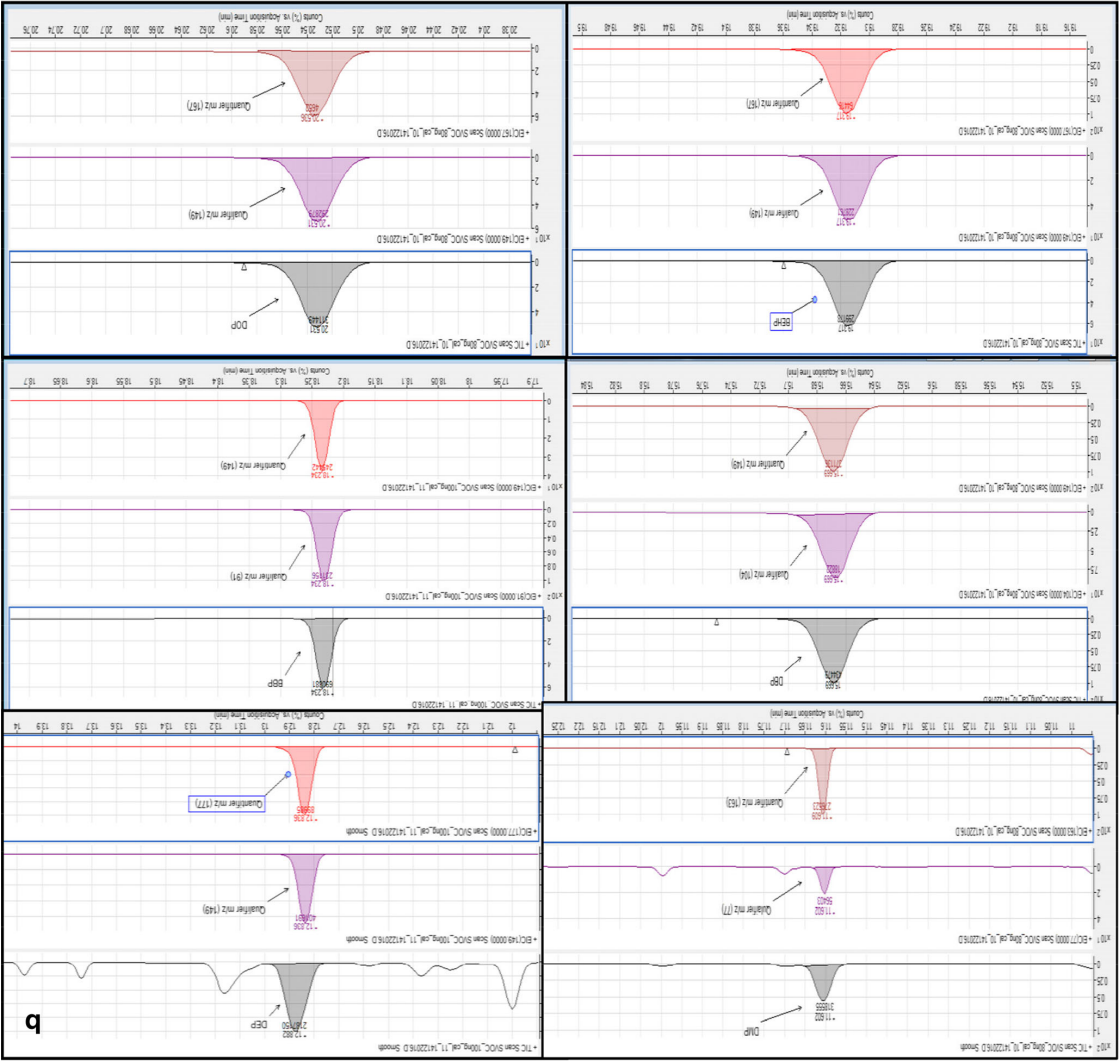
**a** various phthalate standards ion chromatogram obtained GC-MS and **b** Retention time of the six tested phthalate esters obtained by GC-MS

The occurrence and concentration of various phthalate esters, namely DMP, DEP, DBP, BBP, DEHP, and DNOP commonly found in different brands of PET-bottled water were investigated. Because of the high consumption of bottled waters in the Qatari market, assessing the contamination of phthalates is very crucial. Analysis was done to determine the factors affecting the leaching of phthalates from PET bottles into the water. Figure 2 illustrates the various phthalate standards ion chromatogram obtained GC-MS and the retention time of the six tested phthalate esters obtained by GC-MS.

### Concentration of phthalate esters stored at 30 °C

Various organizations had set certain limits for the concentrations and levels of phthalates in drinking water due to their harmful effect on human health. According to the EU Council [17], the DEHP and BBP maximum contaminant levels (MCL) are 0.006 μg/mL and 1 × 10^−4^ μg/mL, respectively. While the threshold limit values (TLV) for DEP, DBP, DMP, and DEHP are 0.55 μg/mL, 0.45 μg/mL, 5.0 μg/mL, and 5.0 μg/mL, respectively.

Table 2 presents the concentration range of various phthalate esters (μg/mL) reported from different PET bottled water samples. The detected level of the BBP was more than the MCL in only one sample out of six, while the DEHP was detected at a concentration higher than the MCL in four water samples, but lower than the TLV in all samples. However, the DMP, DBP, and DEP were found to have lower levels than the TLV. The DNOP was not detected in any of the samples except for one sample. On the other hand, the DMP was the most abundant phthalate compound that was detected in all samples at low concentrations ranging between 0.001–0.05 μg/mL, and the DEP was the second abundant compound and found in 5 samples out of 6 at concentrations ranging between 0.008–0.23 μg/mL. These results were also compared with other available data in the literature as shown in Table 2.

**Table 2:**
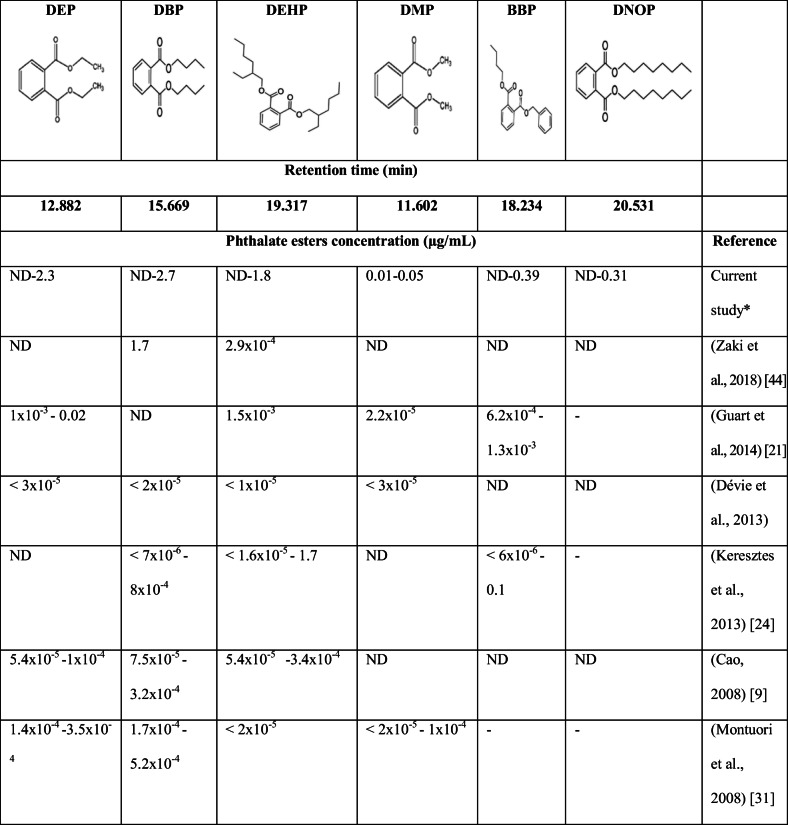
Concentration range of various phthalate esters (μg/mL) reported from different PET bottled water samples

ND indicates that compounds were not detected, and dashed lines are listed when data is not available. LOD in mg/L for the current study: DMP = 0.01, DEP = 0.009, DBP = 0.006, DEHP = 0.009, DNOP = 0.009. Experimental conditions for the current study: temperature 30 °C; pH: >11; storage time: ≈ 48 h

It can be concluded that our results are higher than the previously published data. This could be attributed to the fact that the previous studies examined phthalate levels in PET-bottled water directly after production or purchase, but in our study, the phthalates were investigated after 48 h. The common phthalates reported in several bottled water samples were DBP and DEHP. However, the results for the BBP were consistent with the results obtained by Jeddi et al. [22], who found that it was not detected in the tested samples stored at low temperatures (25–30 °C). Moreover, Domínguez-Morueco et al. [12] examined DEP and DBP levels in bottled water and found that their concentrations were 0.011 and 0.91 μg/mL, respectively, in which DEP results are consistent with our results to some extent. However, the DBP levels were above our obtained values. This variation in the concentration of different phthalates between this current study and others might originate from the differences in contaminant occurrence in different regions [15]. In Qatar, the bottled water is predominantly natural groundwater and from the desalination units. Bono-Blay et al. [8] reported that phthalates could percolate down and reach the aquifers deeply. Furthermore, Bach et al. [7] reported that industrial and municipal activities could present possible contributing factors for landfill leachate. According to the European Commission [16], the specific migration limit (SML) for DBP and BBP was 0.3 and 30 mg/kg, respectively, which are higher than our obtained results.

### Effect of storage temperature on phthalate esters leaching

**Fig. 3:**
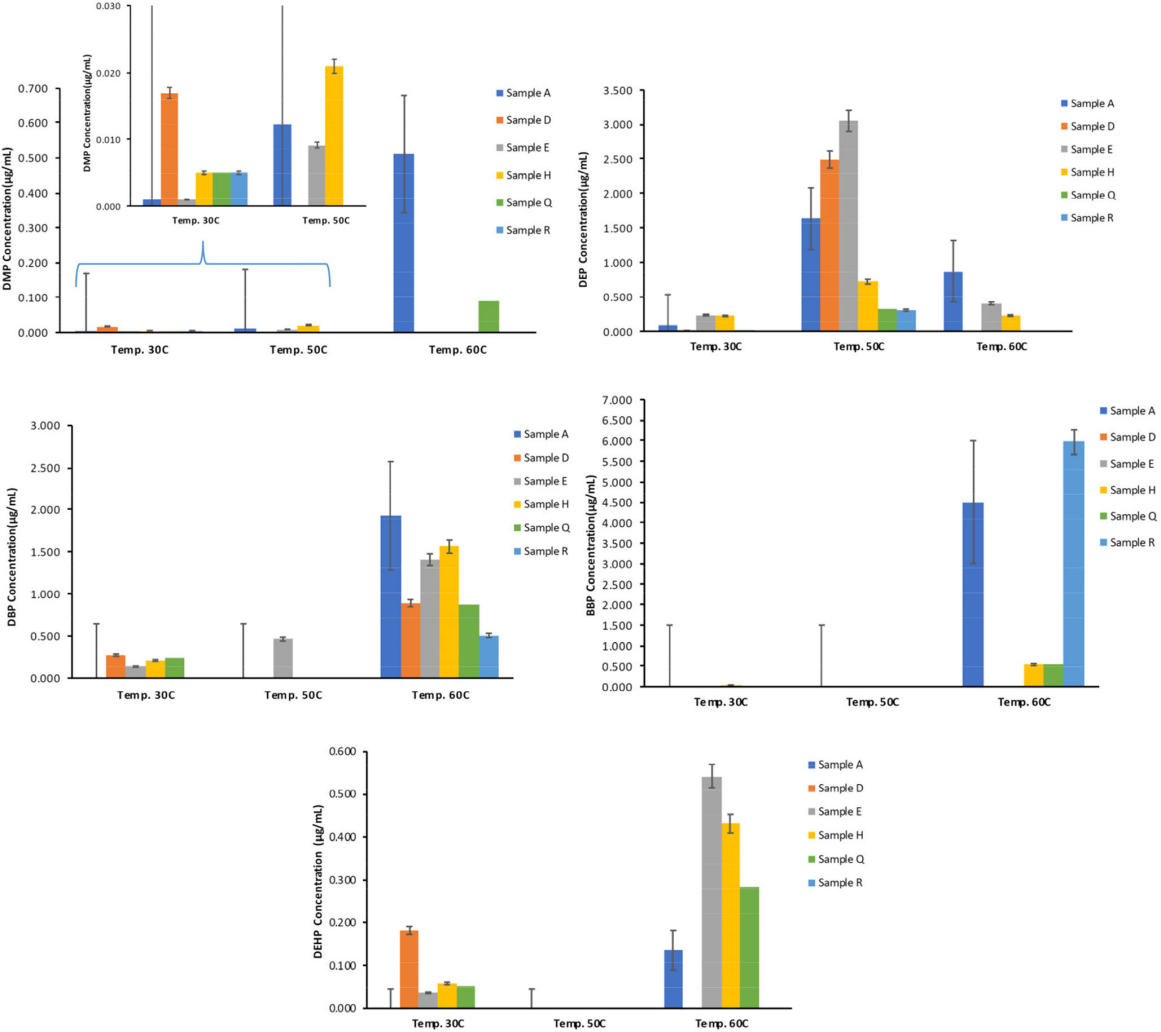
Average concentration (μg/mL) of phthalates present in different brands of PET-bottled drinking water stored in oven at various temperatures: **a**: 30 °C, **b**: 50 °C, and **c**: 60 °C for 48 h

The levels of the phthalates were examined in various water samples after being stored at different temperatures (30 °C, 50 °C, and 60 °C) for 48 h. Figure 3 shows the average concentration (μg/mL) of phthalates (DMP, DEP, and DBP) present in different brands of PET-bottled drinking water stored in an oven at various temperatures: A: 30 °C, B: 50 °C, and C: 60 °C, for 48 h. The obtained results showed an increasing trend of the concentration of all phthalates present in the tested water samples with increasing the storage temperature to 60 °C.

Among the six analyzed drinking water samples, the DNOP was not detected in any sample, while the DEHP was detected in four samples at 30 °C at the concentration level of 0.037–0.182 μg/mL. The DEHP was not detected in any sample when the temperature increased to 50 °C, and with increasing the temperature to 60 °C, it was detected in five samples with concentration ranging between 0.02 μg/mL - 1.3 μg/mL. These results are higher than the MCL for the DEHP of 0.006 μg/mL and the TLV of 0.005 μg/mL. Guart et al. [20] studied the effect of temperature (> 40 °C) on the migration of DEHP from plastic bottles to water and found that there are no detected traces of DEHP. Amiridou and Voutsa [6] found that the median concentration of DEHP in PET-bottled water was 3.5 × 10^−5^ μg/mL, while Montuori et al. [31] found that the concentration of DEHP was 2 × 10^−5^ μg/mL. Besides, BBP was detected in only one sample at 30 °C with a concentration of 0.039 μg/mL, and it was not detected in any sample at 50 °C, which is consistent with results found by Ceretti et al. [11], who found no traces of BBP in the studied water samples, which were incubated at 50 °C. However, the BBP was detected in four samples out of six at the concentration level of 1.05 μg/mL - 11.9 μg/mL in which these concentrations are higher than the maximum contaminant level (MCL) of 0.001 μg/mL [17]. Furthermore, the results obtained by Casajuana and Lacorte [10], who studied the effect of temperatures higher than 40 °C on water samples that were not analyzed directly after purchasing and found that the maximum detected concentration of BBP was 0.13 μg/L which is less than our results. According to Al-Saleh et al. [5], higher BBP concentration was found in water samples incubated at 60 °C than at 30 °C, as well as higher than the limit of qualification (LOQ) of 0.994 μg/L and MCL of 0.1 μg/L.

The same trend was followed with the DBP, as it was detected only in one sample at 50 °C with a concentration of 0.46 μg/mL. Moreover, it was detected in four samples at 30 °C within the range of 0.137 μg/mL - 0.242 μg/mL and in all samples at 60 °C within the concentration range of 0.51 μg/mL - 1.93 μg/mL. Although the concentration of almost all phthalates in most samples decreased at temperature 50 °C and then increased with increasing the temperature to 60 °C, the concentration of DEP increased with increasing the temperature from 30 °C to 50 °C and decreased to no detectable level with increasing the temperature to 60 °C. The DEP and DMP were detected in all samples at 30 °C. However, at 50 °C, the DEP was detected in all samples while the DMP was only detected in three samples at a concentration level of 0.3 μg/mL - 2.49 μg/mL and 0.01 μg/mL - 0.02 μg/mL, respectively. Furthermore, increasing the temperature to 60 °C did not have the same effect on both DEP and DMP where DEP was detected only in 3 samples, and DMP was detected in 5 samples, with a concentration range of 0.22 μg/mL - 0.58 μg/mL and 0.002 μg/mL - 0.51 μg/mL, respectively. These results are not consistent with the results obtained by Salazar-Beltrán et al. [35], in which they got lower concentrations for DBP (0.02 μg/mL - 0.08 μg/mL) and DMP was only detected in one of the ten analyzed samples with a concentration of 0.003 μg/mL. Kanchanamayoon et al. [23] investigated the presence of various phthalate esters in different PET-bottled water brands exposed to 60 °C and found that DMP, DEP, and DBP were only detected in one sample out of five at the level of 0.38 μg/mL, 0.54 μg/mL, and 0.17 μg/mL, respectively. While DEHP was detected in two samples at a concentration of 0.5 μg/mL and 0.28 μg/mL. According to a study conducted by Singh and Li [39], DBP, BBP, and DEHP are reported as the top three phthalates in cardiotoxicity, hepatotoxicity, and nephrotoxicity categories of toxicity. Furthermore, Zaater et al. [43] investigated the presence of phthalates in seven different bottled-water brands from Jordan under room temperature and 50 °C, the results indicated that the tested water brands were contaminated with DBP, DEHP, and DNOP with a total concentration of phthalates under room temperature of 0.0031 μg/mL - 0.020 μg/mL. Moreover, this study reported that increasing the storage temperature to 50 °C caused an increase in the levels of phthalate to ~0.023 μg/mL - 0.03 μg/mL. As demonstrated by various studies, storage duration, sunlight and temperature change are the reason behind the presence of DEHP in bottled water, although there are no convincing explanations since the origin of DEHP can include several different things such as cap-sealing resins, background contamination, and PET containers.

### Effect of remediation by various adsorbents

Table 3 shows the final concentration of DEP, DBP, and DEHP after remediation with different masses of AC, RODP, and S-RODP, respectively. The remediation studies were carried out for the samples A, D, E, H, and Q. R had a none detectable concentrations of DEP, DBP, and DEHP at 30 °C.

**Table 3:** Concentration and standard deviation (SD) of DEP, DBP, and DEHP in (μg/mL) after treatment of phthalates contaminated drinking water applying different amounts of AC, RODP, and S-RODP at 30 °C

Phthalate esters μg/mL (×10^−3^)	A			D			E			H			Q		
	Adsorbent mass (g)														
	0.05	0.1	0.15	0.05	0.1	0.15	0.05	0.1	0.15	0.05	0.1	0.15	0.05	0.1	0.15
AC															
DEP	7.76	ND*	ND	6.29	4.59	1.94	0.97	0.48	ND	4.5	1.11	1.0	2.04	1.5	0.96
SD	0.051	–	–	0.07	0.06	0.032	0.015	0.01	–	0.12	0.05	0.01	0.05	0.1	0.01
DBP	5.1	1.9	1.9	7.2	4.9	4.9	1.5	1.4	1.4	4.9	3.2	1.5	1.6	1.1	0.96
SD	0.1	0.05	0.05	0.2	0.1	0.1	0.04	0.04	0.04	0.1	0.2	0.04	0.04	0.05	0.01
DEHP	1.3	0.56	ND	6.8	1.1	0.8	3.7	0.9	0.3	2.0	1.11	0.73	0.085	ND	ND
SD	0.1	0.02	–	0.15	0.05	0.07	0.09	0.01	0.01	0.1	0.01	0.02	0.003	–	–
RODP															
DEP	0.36	ND	ND	0.23	ND	ND	2.35	0.73	0.43	4.5	1.11	1.0	2.04	1.5	0.96
SD	0.006	–	–	0.02	–	–	0.01	0.02	0.02	0.12	0.01	0.06	0.03	0.01	0.02
DBP	0.65	ND	ND	2.8	2.0	1.8	4.1	2.7	0.42	0.4	0.2	0.1	0.1	ND	ND
SD	0.02	–	–	0.1	0.1	0.1	0.15	0.2	0.025	0.02	0.015	0.02	0.02	–	–
DEHP	0.13	ND	ND	1.3	0.7	0.55	3.7	0.9	0.3	ND	ND	ND	ND	ND	ND
SD	0.02	–	–	0.25	0.15	0.02	0.2	0.02	0.015	–	–	–	–	–	–
S-RODP															
DEP	5.9	ND	ND	2.5	ND	ND	ND	ND	ND	ND	ND	ND	0.67	0.30	ND
SD	0.15	–	–	0.25	–	–	–	–	–	–	–	–	0.01	0.02	–
DBP	0.33	ND	ND	0.65	0.28	1.3	0.23	ND	ND	1.0	0.54	ND	ND	ND	ND
SD	0.02	–	–	0.03	0.02	0.02	0.02	–	–	0.07	0.04	–	–	–	–
DEHP	0.04	ND	0.07	ND	ND	ND	ND	ND	ND	ND	ND	ND	ND	ND	ND
SD	0.005	–	0.001	–	–	–	–	–	–	–	–	–	–	–	–

LOD in mg/L: DEP = 0.009, DEHP = 0.009. Experimental conditions: volume of solution: 100 mL; TEMP. 30 °C; pH: 8; contact time: 72 h

It was noticed that most of the phthalates were not detected in any tested sample after using the S-RODP. It was also observed that increasing the mass of the adsorbent leads to a decrease in the phthalate concentrations. The removal percentage of an adsorbent was calculated using eq. 2. Furthermore, from the obtained results, it can be concluded that S-RODP has the highest removal capacity than other adsorbents due to the newly formed functional groups on the surface of the adsorbent because of the applied chemical modifications.

2 $$ \left[\frac{\Big({C}_{{}^{\circ}}-{C}_e}{C_{{}^{\circ}}}\right]x\ 100 $$

Where, C˳ is the average concentration (μg/mL) and C_e_ is the equilibrium average concentration (μg/mL).

### Effect of temperature on the adsorption of phthalates by S-RODP

**Fig. 4:**
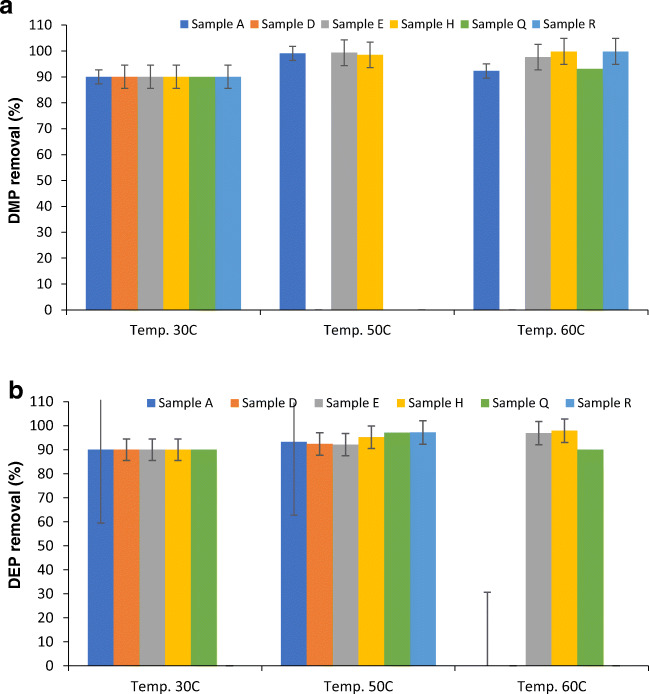

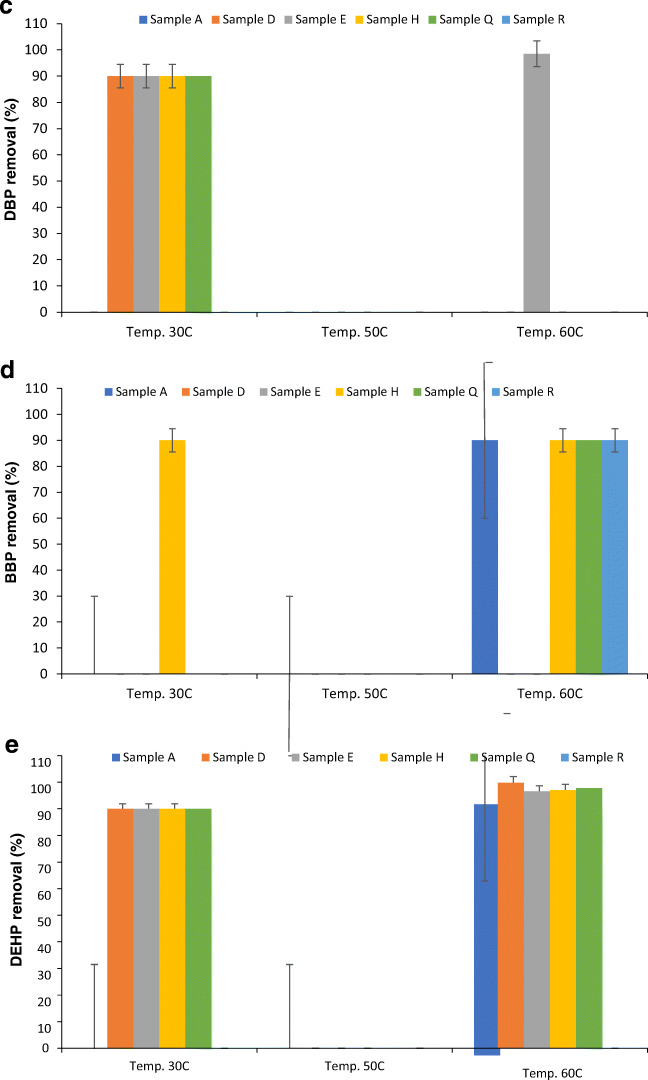
Removal percentage of (**a**) Dimethyl phthalate (DMP), (**b**) Diethyl phthalate (DEP), (**c**) Dibutyl phthalate (DBP), (**d**) Benzyl butyl phthalate (BBP), and (**e**) Bis(2-ethylhexyl) phthalate (BEHP) after extraction from plastic bottled water heated in the oven at different temperatures (30 °C, 50 °C, 60 °C) from the collected water samples using S-RODP

As shown in Fig. 4a, the removal percentage was increased with increasing the temperature for samples H, Q & R; while for sample A, it was increased from 90% to 99% by increasing the temperature from 30 °C to 50 °C and then decreased to 92.3% at 60 °C. Dimethyl phthalate (DMP) concentrations decreased with increasing temperature for all water samples except for sample A and sample Q. It was increased from 0.001 μg/mL for temperature 30 °C to 0.51 μg/mL for temperature 60 °C for sample A, and from 0.005 μg/mL to 0.32 μg/mL when temperature increased from 30 °C to 60 °C. However, the results in Fig. 4b shows that the removal percentage was increased with increasing the temperature for samples A, D, E, & H, while samples Q and R observed fluctuation in the removal percentage with increasing the temperature from 30 °C to 60 °C. Furthermore, the concentrations of diethyl phthalate (DEP) were increased with increasing the temperature from 30 °C to 50 °C, but it either decreased or not detected when the temperature increased from 50 °C to 60 °C. Moreover, as shown in Fig. 4c, 90% was the removal percentage for samples D, E, H, & Q at 30 °C and it was increased to 98.5% for sample E at 60 °C. Furthermore, dibutyl phthalate (DBP) was not detected in all water samples when the temperature increased from 30 °C to 50 °C, except for water sample E. The concentration was increased from 0.14 μg/mL to 0.46 μg/mL, but when the temperature increased to 60 °C the concentration of the DBP increased for all water samples in which it all falls in the range of 0.51 μg/mL for the sample R to 1.93 μg/mL for the sample A. These results agree with other previously published studies [1].

As shown in Fig. 4d, the removal percentage was almost the same for all water samples. Furthermore, BBP was not detected in any water sample when the temperature was increased from 30 °C to 50 °C. It was only detected in the sample H under 30 °C (0.039 μg/mL). According to Ceretti et al. [11], who studied the presence of different phthalates, including BBP in water samples incubated at 50 °C and they found no traces of BBP. However, this was not the case when the temperature increased to 60 °C, the BBP was detected in the samples A, H, Q & R as follows 8.9 μg/mL, 1.06 μg/mL, 1.05 μg/mL, and 11.9 μg/mL, respectively. These concentrations were higher than the maximum contaminant level (MCL) of 0.1 μg/L (EU Council [17]). A study was conducted in Saudi Arabia to determine phthalate residues in different water samples stored under different conditions including 4 °C, 30 °C, and 60 °C. It was found that the maximum levels of BBP were found in water samples stored under 4 °C and followed by 60 °C and the lowest were found under 30 °C. Besides, the BBP concentrations were detected in 76% of all tested bottled water samples and were exceeded the limit of qualification (LOQ) of 0.994 μg/L and the MCL of 0.1 μg/L [5]. Furthermore, the obtained results were higher than the results found by Casajuana and Lacorte [10].

According to a study conducted by Singh and Li [39], DBP, BBP, and DEHP were reported as the top three phthalates in cardiotoxicity, hepatotoxicity, and nephrotoxicity categories of toxicity. As demonstrated by various studies, storage duration, sunlight and temperature change are the reason behind the presence of DEHP in bottled water, although there are no convincing explanations since the origin of DEHP can include several different things such as cap-sealing resins, background contamination, and PET containers [7]. As shown in Fig. 4e, the removal percentage for all tested water samples was increased by increasing the temperature from 30 °C to 60 °C. Moreover, likewise, BBP, increasing the temperature from 30 °C to 50 °C did not increase the concentration of DEHP and it was not detected in any sample. These results agreed with the results found by Guart et al. [20], who studied the effect of temperature (>40 °C) on the migration of DEHP from plastic bottles to the water and found that there are no detected traces of DEHP.

On the other hand, further increase in the temperature to 60 °C leads to the detection of DEHP in the samples A, D, E, H, and Q with concentrations of 1.30 μg/mL, 0.02 μg/mL, 0.54 μg/mL, 0.43 μg/mL, and 0.28 μg/mL, respectively. As mentioned previously, the MCL for DEHP is 6 μg/L and the TLV is 5.0 mg/L which means that the obtained results were below these values. Amiridou and Voutsa [6] studied the effect of temperature on the concentration of DEHP and found that the mean concentration was 0.350 μg/L. Nevertheless, there were no detectable traces of DOP in any tested water sample. Additionally, as mentioned by previous studies, it is difficult to observe a net effect of each one of the different storage conditions of phthalate migration due to the missing measurements of the initial phthalate levels before and after storage ([24]; Casajuana and Lacorte [10]. Hence, our findings showed that the observed increase in the concentration of phthalate was due to the increase in the temperature causing the migration of phthalate from the PET materials.

### Mechanism of adsorption: Structural analysis characterization by Fourier transform infrared spectroscopy (FTIR)

The surface chemistry of the adsorbent was examined using FTIR. This will help in understanding the adsorption mechanisms of the phthalates onto the adsorbent surface and their effects on the adsorption process. The phthalates present in bottled waters are of varying chemical structures and physicochemical properties and as a result, interact differently with various types of adsorbents. Adsorption driving force is a key factor in determining the adsorption mechanisms. According to Akpomie et al. [1], the first driving force for the adsorption process is the solute solubility and its relationship with the solvent. Moreover, the second driving force is the solute affinity for the adsorbent surface that is affected by the adsorbent surface characteristics. Therefore, the surface chemistry of the adsorbents and their effect on the adsorption processes were described to interpret the phthalates adsorption onto various adsorbents.

**Fig. 5:**
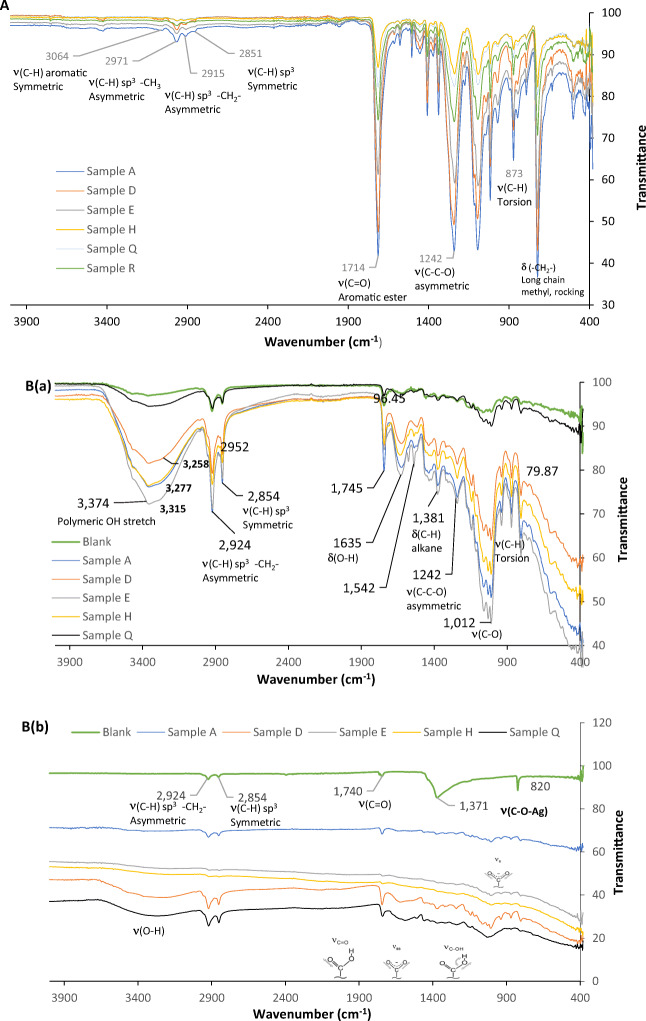
**a** FTIR spectra showing various peaks corresponding to different functional groups representing the water bottle composition of different water brands, and **b** FTIR spectra of (*a*) RODP, and (*b*) S-RODP before and after phthalates adsorption from various tested water samples. Experimental conditions: volume of solution 100 mL; pH 8; temperature 30 °C; contact time 72 h; adsorbent mass 0.05 g

FTIR is used to investigate the interaction between adsorbate and the adsorbent’s active sites, and it can determine the functional groups that are responsible for the adsorption process. The FTIR measurements were performed over 4000–400 cm^−1^. The physical characteristics of the PET material in the tested bottles were analyzed to determine the interaction between the packaging material and the water. Figure 5a illustrates the functional groups found in the PET bottles of various water brands, in which four functional groups are commonly found on all tested bottles detected at 1714 cm^−1^ as the highest peak corresponding to the primary C=O bond (aromatic ester), followed by 1242 cm^−1^ corresponding to an asymmetric C-C-O stretching bond involving an aromatic ring. Furthermore, 1097 cm^−1^ and 722 cm^−1^ were found in the fingerprint region determining the presence of Si-O-Si and the aromatic C-H wagging, respectively [38, 41]. Our results are consistent with results found by Mohamed et al. [29], who investigated the structural changes and PET strength in plastic bottles after being exposed to various storing conditions and he reported that the presence of C=O bond is due to the aldehyde structure in the PET as the main structure.

To characterize the interaction between the phthalate esters with the RODP and the S-RODP, the different adsorbents were analyzed by FTIR before and after the adsorption process. Firstly, the characteristic peaks were observed on two regions on the surface of the adsorbents from 3350 to 1750 cm^−1^ and the second region was below 1750 cm^−1^. More than one peak was observed at the surface of the RODP and the S-RODP including peaks at 3064, 2971, 2915 cm^−1^ and 2851 cm^−1^ corresponding to (C-H) aromatic, (C-H, -CH_3_) sp^3^ asymmetric, (C-H, -CH_2_-) sp^3^ asymmetric and (C-H, -CH_3_) sp^3^ symmetric peak, and at 1714 cm^−1^ indicating a C=O stretch (aromatic ester). The 1242 cm^−1^ was attributed to the C-C-O asymmetric.

Comparing the functional groups present on the surface of the RODP before and after adsorption of phthalate esters, the adsorption process caused a slight change in the FTIR spectra as shown in The FTIR spectrum of the adsorption of phthalate esters (pH of the water sample = 8) has a complex band at 700–1800 cm^−1^ (Fig. 5b(*a*)). Multiple new bands were formed after the adsorption process, including a peak at 3369 cm^−1^ that appeared after the adsorption of multiple water samples corresponding to a carboxylic group, another peak was at 1624 cm^−1^ refereeing to 1°amines (N-H bend), aliphatic amine (C-N stretch) at 1248 cm^−1^ and C-H bending was observed at 1380 cm^−1^ representing alkane group. It could be concluded that the responsible functional groups for phthalate adsorption on the surface of RODP are carboxylic and alkane groups. The functional groups formed on the surface of RDP are one strong broad peak at the region of 3374 cm^−1^ ascribing the presence of stretching vibrations of OH. Another peak was found at 1635 cm^−1^, which indicates the OH bending of absorbed water. Peaks at 1381 and 1012 cm^−1^ corresponds to the presence of alkanes (C-H rock) in-the-plane CH bending and strong C-C C-OH, C-H ring, and side group vibrations, respectively.

This fact also confirms our conclusion that the abroad peak at 3474 cm^−1^ belongs to –OH groups vibrations in the date pits (cellulose), located near phthalate esters molecules and promoting the H-bond bridge formation, including dimerization process. The phthalate esters molecules deprotonation and dimer destruction follow to changing the interaction character with –OH groups in water molecules. According to the spectrum, H-bond strength increases. It is manifested in the hypsochromic shift of the band from 3000 cm^−1^ –3626 cm^−1^ to 3059 cm^−1^ –3551 cm^−1^.

In the high-frequency spectrum region, a broadband with a peak at 3474 cm^−1^ and a feature in the region of 3258 cm^−1^ was found. These bands belong to –OH groups vibration, involved in H-bonds. At the same time, the peak at 3258 cm^−1^ belongs to the hydrogen bonding vibration participating in the formation of mono- and di-phthalate esters with cellulose. This hypothesis is supported by the disappearance of the first band (3258 cm^−1^). In addition, at 3277 cm^−1^ in the FTIR spectra of more hydrogen bond formation. Apparently, the H-bond formation affects these vibrations symmetry and their group character. The appearance of two modes belonging to the stretching vibrations of OH-bonded groups is observed.

Furthermore, there is a variation in the functional groups present on the surface of S-RODP after the adsorption of different bottled-water brands as shown in Fig. 5b(*b*), in which the main functional groups found on Sample A, are carboxylic acids (O-H stretching and C=O stretching) and carboxylic acid, esters, ethers (C-O stretch), while for Samples E and H, weak peaks were the only peaks in which they were found at the region of 1004 cm^−1^ – 868 cm^−1^ and 1002 cm^−1^, respectively. On the other hand, several common peaks were found after adsorption of phthalate from Samples D and Q in which the main functional group is a carboxylic acid.

**Fig. 6:**
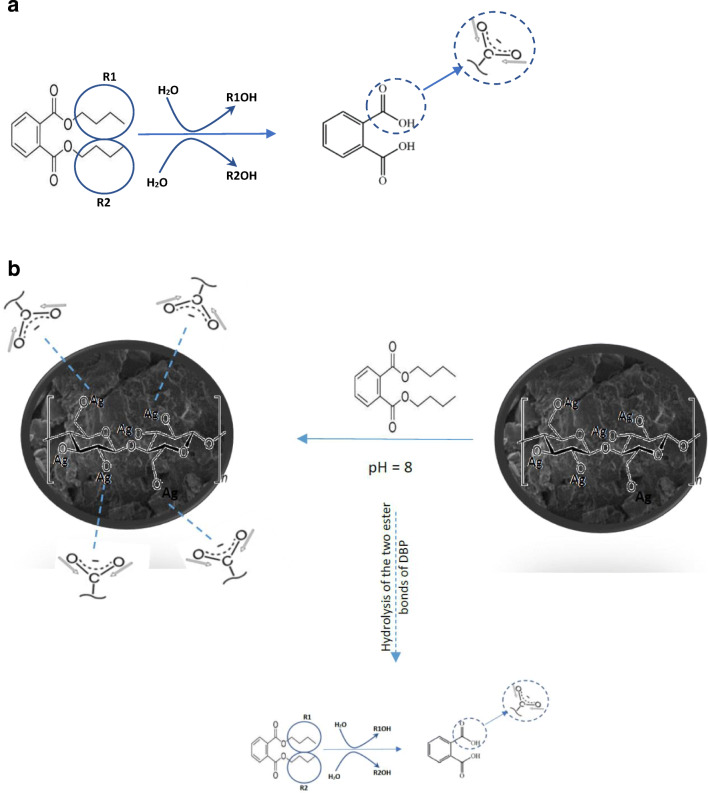

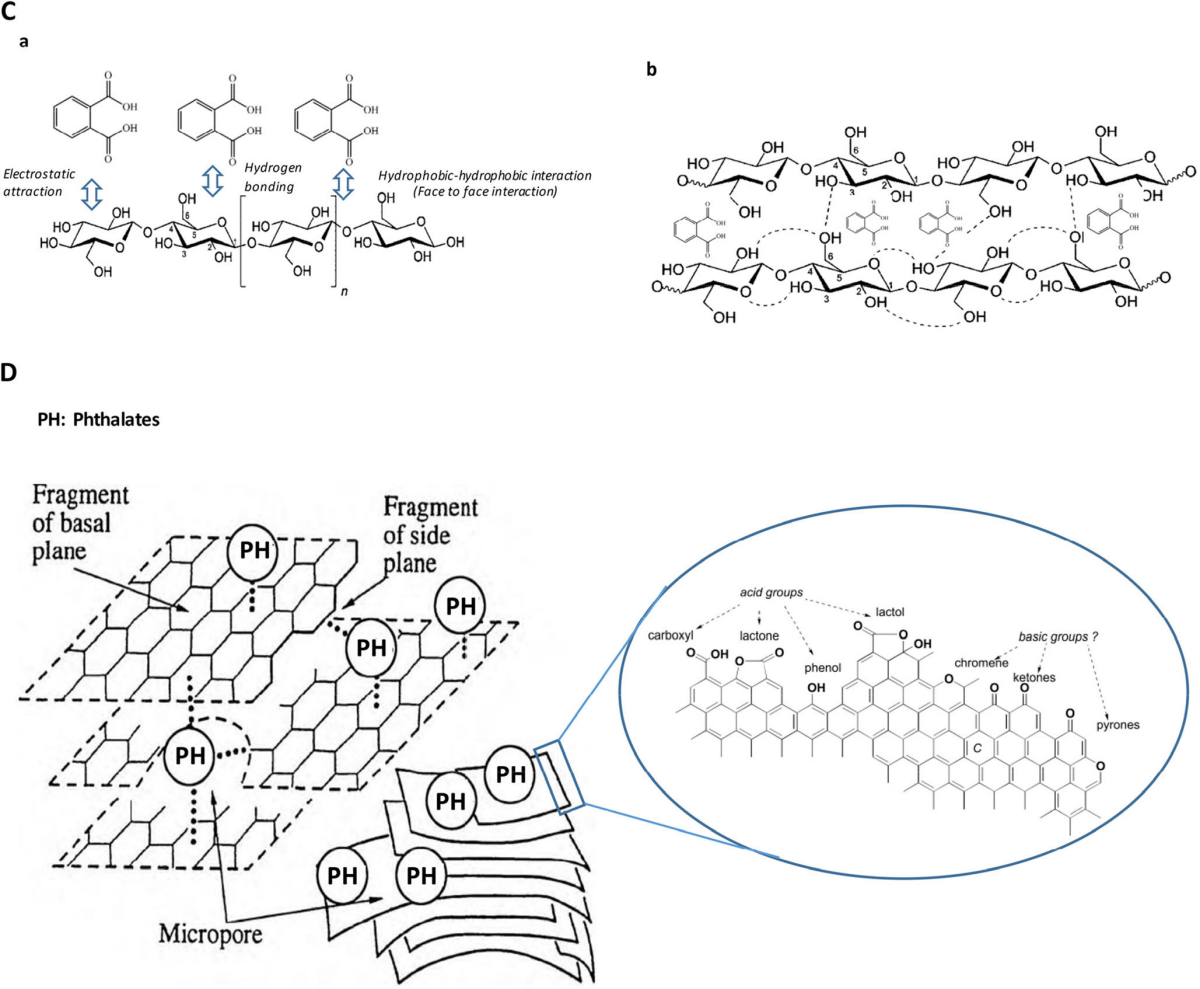
**a** The proposed pathway of DBP hydrolysis and **b** Schematic diagram of the adsorption of DBP onto the S-RPDP, **c** Proposed adsorption mechanism of phthalates onto RODP [34], and **d** Proposed adsorption mechanisms of phthalates onto AC [2, 30]

A carboxyl group gives two main absorption features based on the protonation state of the carboxyl namely carbonyl stretch (*ν*_C=O_) between 1690 cm^−1^ and 1750 cm^−1^, and C-OH vibrations (*ν*_C-OH_) between 1200 cm^−1^ and 1300 cm^−1^. On deprotonation, *ν*_C=O_ shifts to lower energy as its vibrational mode becomes coupled to that of the other oxygen, giving rise to an asymmetric feature (*ν*_as_) between 1540 cm^−1^ and 1650 cm^−1^. Similarly, the C-OH band shifts to higher energy on deprotonation, yielding a symmetric COO- mode (*ν*_s_) between 1300 cm^−1^ and 1420 cm^−1^. As shown in Fig. 5b(*b*), the S-RPDP enhanced the hydrolysis of the two ester bonds of DBP to produce phthalic acid (PA) (Fig. 6a). Figure 6b shows the schematic diagram of the adsorption of DBP onto the S-RPDP.

Figures 6c (*a*)&(*b*) propose possible interaction mechanisms between DBP and the cellulose structures. Different bonding could be proposed such as hydrogen bonding, electrostatic attraction, and hydrophobic-hydrophobic interaction (face-to-face interaction). As mentioned earlier that the surface of RODP is rich in OH and carboxylic (–COOH) groups, which facilitate the formation of hydrogen bonding. In addition, the intra- and intermolecular hydrogen bonds in cellulose would also facilitate the DBP adsorption by RODP (Fig. 6c(*b*)).

The phthalates adsorption onto AC could be attributed to the adsorption of the phthalates by polar functional groups of the AC and to the π-π interactions that developed between cyclic phthalates and the sheets of the AC, as shown in Fig. 6d. The primary functional groups like carboxyl, carbonyl, phenols, lactones, and quinones onto the AC surface are essential for the removal of phthalates. AC includes acidic and basics functional groups that are located on the outer surfaces or edges of the basal plane. Such groups play a major role in the adsorption of various pollutants and have a great influence on the adsorption capabilities of AC [30].

### Statistical analysis

Studying the effect of temperature on the concentration of phthalates was a single factor experiment in which pH was constant throughout the experiment; ANOVA for single factor was used. The smaller the magnitude of *P* values and the larger the magnitude of the F-test value, the higher the significance of the corresponding coefficient. As shown in the following Table 4 that the results are not significantly different at P value ≥0.05.

**Table 4:** One-way ANOVA test for the different adsorbents (AC, RDP, and S-RODP) showing the effect of temperature on different tested phthalates (DEP, DBP, and DEHP)

Source of variation	SS	df	MS	F	P value	F crit
DEP on AC						
Between Groups	6.533333	1	6.533333	0.008031	0.92923	4.195972
Within Groups	22,778.25	28	813.5088			
Total	22,784.78	29				
DEP on RDP						
Between Groups	0.588	1	0.588	0.008324	0.927955	4.195972
Within Groups	1977.887	28	70.63881			
Total	1978.475	29				
DEP on S-RODP						
Between Groups	222.7556	1	222.7556	0.281676	0.603923	4.60011
Within Groups	11,071.51	14	790.8224			
Total	11,294.27	15				
DBP on AC						
Between Groups	47.37633	1	47.37633	0.099934	0.754253	4.195972
Within Groups	13,274.1	28	474.0749			
Total	13,321.47	29				
DBP on RDP						
Between Groups	2.296333	1	2.296333	0.012692	0.911104	4.195972
Within Groups	5065.837	28	180.9228			
Total	5068.134	29				
DBP on S-RODP						
Between Groups	1.12	1	1.12	0.034947	0.853158	4.225201
Within Groups	833.2643	26	32.04863			
Total	834.3843	27				
DEHP on AC						
Between Groups	13.467	1	13.467	0.039676	0.843555	4.195972
Within Groups	9503.947	28	339.4267			
Total	9517.414	29				
DEHP on RDP						
Between Groups	0.54	1	0.54	0.020629	0.887102	4.30095
Within Groups	575.8933	22	26.17697			
Total	576.4333	23				
DEHP on S-RODP						
Between Groups	1.260006	1	1.260006	0.033742	0.85689	4.60011
Within Groups	522.7878	14	37.34199			
Total	524.0478	15				

## Research limitations and future research gap

In the literature, there are various methods for the removal of phthalates from contaminated water. Phthalates treatment from water can be done by physiochemical, biological, and advanced oxidation processes. However, these methods are slow and take longer time than do biodegradation. The common disadvantage for most existing treatment technologies is lower efficiency and longer treatment period, in addition to the operational and maintenance cost. Here, the ease of operation, simplicity of design, and high removal efficiency (90–99%) are advantages of adsorption over other techniques. Agricultural wastes such as date pits (DPs) are considered as cost-effective adsorbents compared to AC, as DPs have macrostructure, physical and chemical properties such as insolubility in water, high mechanical strength, chemical stability, economic viability, and zero economic value. The results obtained from this study demonstrate the potential of using RODP as an effective adsorbent for phthalate esters removal from drinking water. However, S-RODP has the highest removal abilities than other adsorbents due to the newly formed functional groups on its surface. The adsorption properties of RODP were enhanced by the impregnation of silver ions. In this respect, the silver oxide possesses many advantages in the adsorption of phthalates from water; allowing phthalates molecules to readily penetrate their structures and be removed easily, namely by acid-base properties, pore structure, high surface area, and pore volume. Using agricultural waste is beneficial for the environment as certain detrimental consequences can be avoided.

A batch adsorption study would not be enough to understand the adsorption capacity of phthalate esters at a larger water quantity. Therefore, future research may be conducted a thorough comprehensive life cycle cost analysis when different combinations of scenarios are considered to determine the most cost-effective combination of chemical dosage in addition to study of fixed-bed column experiments.

## Conclusion

In this paper, the effect of increasing the temperature on phthalate leaching from PET-bottled water was investigated, and then the remediation capability of RODP, S-RODP, and AC were demonstrated to determine their abilities in removing phthalate contaminants from water. Results indicated that the most abundant phthalate was DMP followed by DEP under 30 °C; however, DNOP was not detected in any of the tested water samples, except for one sample under 30 °C with a concentration of 0.031 μg/mL. Besides, the obtained results showed that phthalate leaching to the bottled drinking water is affected by storage temperature, in which the phthalate esters levels were increased with increasing the temperature to 60 °C. Moreover, the results showed that RODP could successfully be used as an effective adsorbent for phthalate esters removal from drinking water, with applying some modifications to RODP to increase its adsorption efficiency by attaining certain functional groups and micro-pore structure, such as using silver nitrate to produce S-RODP. Adsorption efficiency of the various adsorbents was investigated by using different adsorbent masses, and results illustrated that increasing the adsorbent mass decreased the phthalate levels present in the tested water samples. Although all adsorbents have good adsorption capacities, but results showed that S-RODP has higher removal abilities than other adsorbents due to the newly formed functional groups on its surface. Demonstration of FTIR analysis was done to determine the functional groups found on the surface of the adsorbents after the adsorption process and results indicated that AC has no detected functional groups on its surface, while carboxylic acid groups were mainly found on RODP and S-RODP surface.
